# Toll-Like Receptor 2 Attenuates Traumatic Brain Injury-Induced Neural Stem Cell Proliferation in Dentate Gyrus of Rats

**DOI:** 10.1155/2020/9814978

**Published:** 2020-08-17

**Authors:** Xin Zhang, Yue Hei, Wei Bai, Tao Huang, Enming Kang, Huijun Chen, Chuiguang Kong, Yongxiang Yang, Yuqin Ye, Xiaosheng He

**Affiliations:** ^1^Department of Neurosurgery, Xijing Hospital, Air Force Medical University, Xi'an, 710032 Shaanxi, China; ^2^Health Team of PLA 75768 Troops, Xiangyang, 441500 Hubei, China; ^3^Department of Neurosurgery, Tangdu Hospital, Air Force Medical University, Xi'an, 710032 Shaanxi, China

## Abstract

It was not clear how and whether neural stem cells (NSCs) responded to toll-like receptor 2 (TLR2) in the inflammatory environment after traumatic brain injury (TBI). The current study investigated the correlation of TLR2 and NSC proliferation in the dentate gyrus (DG) using the TBI model of rats. Immunofluorescence (IF) was used to observe the expression of BrdU, nestin, and TLR2 in the DG in morphology. Proliferating cells in the DG were labelled by thymidine analog 5-bromo-2-deoxyuridine (BrdU). Three-labelled BrdU, nestin, and DAPI was used for the identification of newly generated NSCs. Western blotting and real-time polymerase chain reaction (PCR) were used to observe the expression of TLR2 from the level of protein and mRNA. We observed that BrdU^+^/nestin^+^/DAPI^+^ cells accounted for 84.30% ± 6.54% among BrdU^+^ cells; BrdU^+^ and nestin^+^ cells in the DG were also TLR2^+^ cells. BrdU^+^ cells and the expression of TLR2 (both protein and mRNA levels) both elevated immediately at 6 hours (h), 24 h, 3 days (d), and 7 d posttrauma and peaked in 3 d. Results indicated that TLR2 was expressed on proliferating cells in the DG (NSCs possibly) and there was a potential correlation between increased TLR2 and proliferated NSCs after TBI. Taken together, these findings suggested that TLR2 was involved in endogenous neurogenesis in the DG after TBI.

## 1. Introduction

Neurogenesis occurs not only in the immature brain but also in the mature brain of mammals. In the adult brain, it is a complicated process including proliferation, differentiation, migration, and integration of neural stem cells (NSCs) into a neural network [1]. It is well known that NSCs exist mainly in two regions of the adult brain—the subgranular zone (SGZ) of the hippocampal dentate gyrus (DG) [2] and the subventricular zone (SVZ) of lateral ventricles [3]. Cells in these regions have potential self-renewal capacity and can turn out to become newborn neurons, microglia, oligodendrocytes, and astrocytes [4]. Such neurogenesis can be activated in some pathological conditions and play a key role in regeneration, repair, and functional recovery of the central nervous system (CNS) [5–8]. Although many studies have demonstrated that endogenous neurogenesis in the adult brain can be regulated by inflammatory reaction after neural trauma [9–11], it is still not clear how the inflammation within the brain influences the process of neurogenesis through NSC proliferation, differentiation, migration, and integration after traumatic brain injury (TBI).

As the essential innate immune receptors and members of the pattern recognition receptor family, TLRs can initiate primary defense mediated by pathogen-associated molecular pattern (PAMP) and endogenous ligand-induced damage-associated molecular pattern (DAMP) in the CNS [12–16]. TLRs can become stimulated by endogenous ligand released through damaged tissue, which comes from TBI, such as heat-shock proteins (HSPs), molecular degradation products, and intracellular components of damaged cells, which are called DAMPs. TLR signaling pathways contain two main parts, which are the MYD88-dependent pathway and the MYD88-independent pathway [17]. The inflammatory reaction caused by TBI can initiate activation of NF-*κ*B and inflammatory cytokine genes. TLR2 is an identified TLR analog in mammals, and evidences have shown that TLR2 can be seen on several brain cells including neurons, microglia, oligodendrocytes, and astrocytes [18–20]. As known, it is important in the identification of an inflammatory environment in CNS-associated disorders, such as brain hemorrhage, infarction, and trauma [21–24]. Activation of TLR2 can mediate activation of NF-*κ*B through the MYD88-independent pathway. Some recent investigations exhibit that the TLR2 expressed on the surface of NSCs and participated in the proliferation and differentiation of NSCs [25–27]. Nevertheless, it is not known what a potential role TLR2 plays in neurogenesis and whether and how NSCs respond to TLR2 in the inflammatory environment in the DG after TBI [28]. The current study observed NSC proliferation and TLR2 expression using a rat TBI model with an aim of exploring the potential correlation of TLR2 and endogenous neurogenesis (mainly NSCs involved) in the DG after TBI.

## 2. Results

### 2.1. Neurological Function Deficiency and Morphological Abnormality

**Figure 1:**
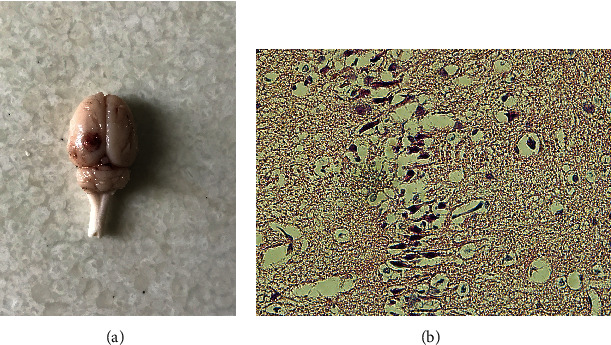
Whole rat brain and injured cortex image. (a) Contusion and bleeding area on the surface at the parietal and occipital brain; (b) a large number of shrink neurons with pyknosis occurred in the injured cortex. Scale bar: 50 *μ*m.

After being performed by controlled cortical impact (CCI), all injured rats were back-arched, hair-erected, and in a rapid and shallow breath. These above manifestations disappeared in 1-2 hours; meanwhile, the rats were kept on the heating plate. It was observed that their right limbs were paralyzed. Sections of cortex containing injury sites 1 day after trauma were prepared for hematoxylin and eosin staining (H&E). Contusion and bleeding were seen on the surface at the parietal and occipital cortex (Figure 1(a)), and pyknosis among lots of shrink neurons was observed under microscopy (Figure 1(b)).

### 2.2. NSC Proliferation

**Figure 2:**
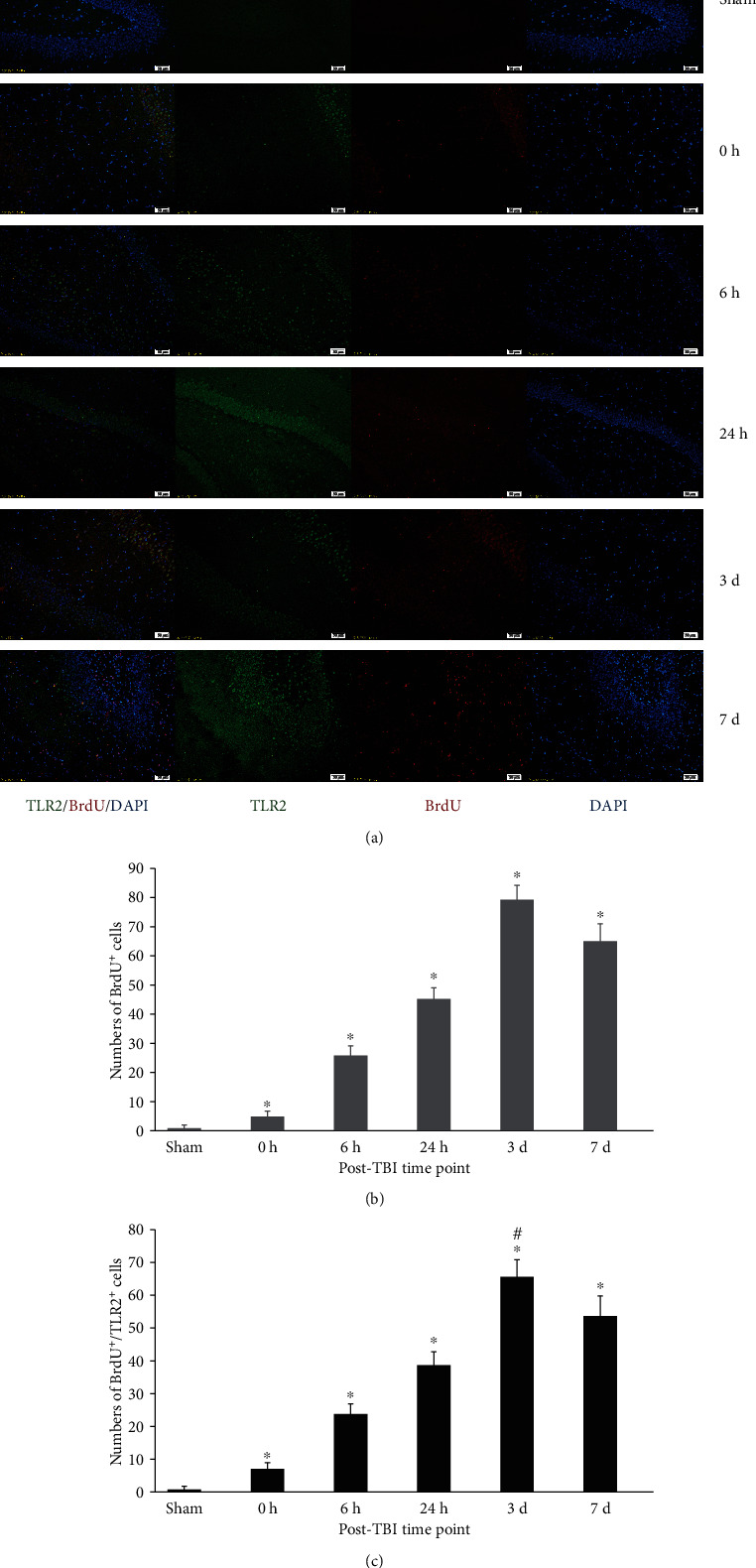
Immunofluorescence (IF) microphotographs in the dentate gyrus (DG) of the DG in sham and TBI groups at different time points. (a) BrdU-labelled NSCs (red fluor), expression of TLR2 (green fluor), cell nuclei (blue fluor), and BrdU^+^/TLR2^+^/DAPI^+^ cells indicated that TLR2 expression in labelled proliferating cells was possible NSCs; (b) BrdU^+^ cells showed the proliferation of NSCs in the DG during different time points posttrauma. BrdU^+^ cells were more in the TBI group than that in the sham group (^∗^*p* < 0.05), and numbers of these cells were significantly different among different time points posttrauma (^#^*p* < 0.05); (c) numbers of BrdU^+^/TLR2^+^ cells indicated that the expression of TLR2 was quite different in proliferating cells of the DG among different time points posttrauma. There were more BrdU^+^ cells in the TBI group than that in the sham group (^∗^*p* < 0.05), and the numbers of these cells are obviously different among different time points posttrauma (^#^*p* < 0.05). Scale bar: 50 *μ*m; data is shown as mean ± SEM.

**Figure 3:**
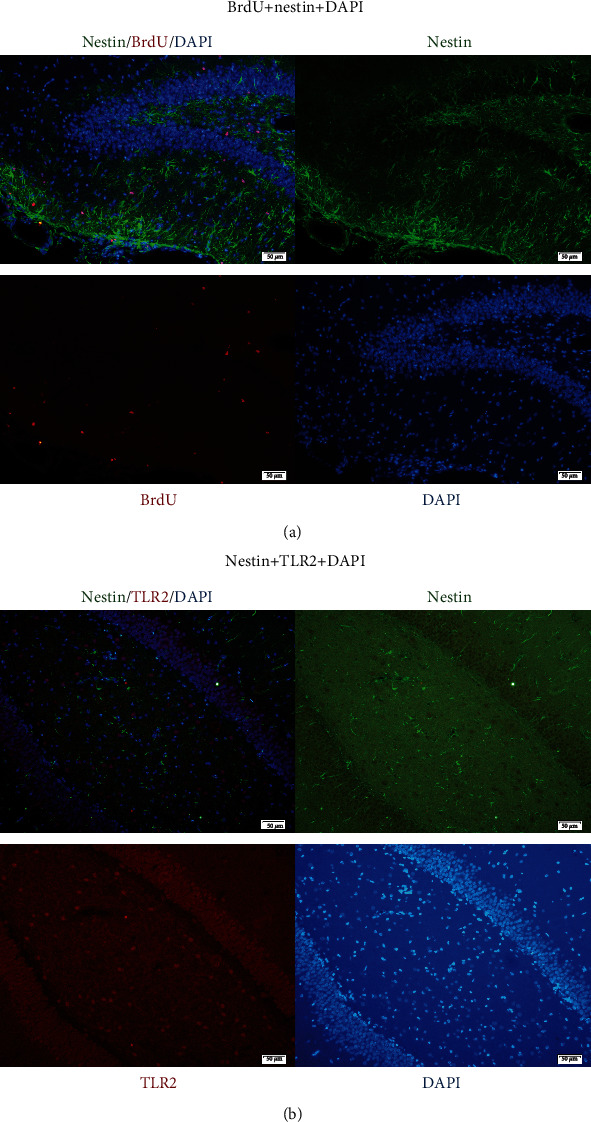
IF in the DG of the TBI group (mouse brain got from 3 days posttrauma). (a) BrdU (red), nestin (green), and DAPI (blue), respectively, exhibited proliferating cells, NSCs, and cell nuclei in the DG. Merged pictures of BrdU^+^/nestin^+^/DAPI^+^ showed NSCs (the percentage of NSCs in proliferating cells was 84.30% ± 6.54%); scale bar: 50 *μ*m; data were expressed as mean ± SEM. (b) Nestin (green), TLR2 (red), and DAPI (blue), respectively, exhibited NSCs, TLR2 expression, and cell nuclei in the DG. Merged pictures of nestin^+^/TLR2^+^/DAPI^+^ showed the expression of TLR2 on NSCs. Scale bar: 50 *μ*m; data were expressed as mean ± SEM.

Compared with the sham group, BrdU-positive cells in the DG increased markedly in rats after TBI (*p* < 0.05). BrdU^+^ cells increased right after brain trauma, reached the peak in the 3rd day, and then decreased gradually (Figure 2(b)). BrdU, nestin, and DAPI three-labelled immunofluorescence (IF) images showed NSC proliferation in the DG. The proliferating cells, NSCs, and cell nuclei were indicated as red fluor (BrdU^+^), green fluor (nestin^+^), and blue fluor (DAPI^+^), respectively. BrdU^+^/nestin^+^/DAPI^+^ cells accounted for 84.30% ± 6.54% among all BrdU^+^ cells (Figure 3(a)). The percentage was consistent with the increasing of proliferating NSCs in the DG.

### 2.3. Expression of IF, Morphology, Protein, and mRNA of TLR2 in the Dentate Gyrus

**Figure 4:**
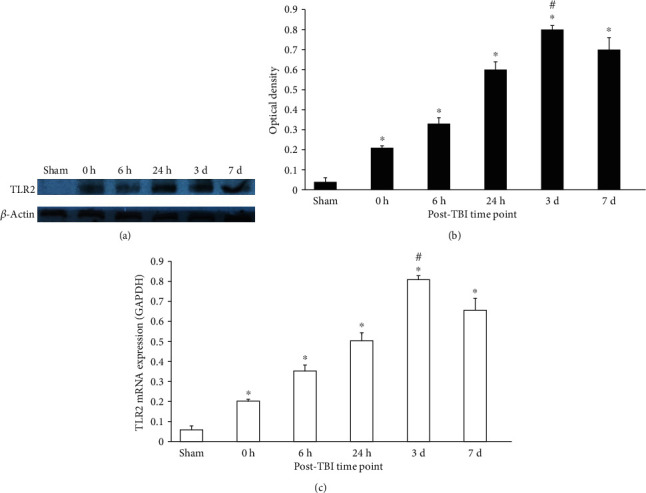
Expression of TLR2 protein and mRNA in the DG (western blotting and PCR). (a) Western blotting: electrophoresis bands of TLR2 protein controlled with *β*-actin; (b) western blotting: the optical density of TLR2 electrophoresis; (c) real-time PCR: the expression of TLR2 mRNA and GAPDH was used as the endogenous reference gene. The TLR2 expression in the protein and mRNA level was significantly higher in the TBI group than that in the sham group (^∗^*p* < 0.05), and the TLR2 expression was significantly different among various time points (^#^*p* < 0.05). Data were expressed as mean ± SEM.

TLR2^+^ cells could be seen with red fluor on cytomembrane in the DG (Figures 2(a) and 3(b)). TLR2^+^/nestin^+^/DAPI^+^ three-labelled fluor showed TLR2 expression on NSCs (Figure 3(b)). BrdU^+^/TLR2^+^/DAPI^+^ three-labelled fluor showed TLR2 expression in proliferating cells (mainly NSCs) in the DG (Figure 2(a)). Quantitative analysis indicated that BrdU^+^/TLR2^+^/DAPI^+^ cells increased immediately after trauma and peaked in the 3rd day, then decreased gradually in the 7th day (Figure 2(c)). The TLR2 protein expression in the DG increased immediately after trauma, peaked in the 3rd day, and decreased in the 7th day posttrauma, but significantly higher compared with sham (*p* < 0.05) (Figures 4(a) and 4(b)). The expression of TLR2 mRNA elevated immediately after trauma, peaked in the 3rd day, and then decreased to a level still significantly higher than in sham (*p* < 0.05) (Figure 4(c)).

## 3. Discussion

Neurogenesis is a continuous process including proliferation, differentiation, and migration of NSCs after TBI [16, 29]. The current study focused on the proliferation process of NSCs. The rats were injected with BrdU intraperitoneally which was used to label newly generated or proliferating cells in the DG [30]. Meanwhile, nestin was expressed on the early stage of NSCs and it was used here to identify the NSCs among all of the proliferating cells in the DG and cortex. The expression of nestin was relatively stable in the proliferation of NSCs; however, it might change in the differentiation of NSCs into neurons, microglia, oligodendrocytes, and astrocytes. The current results revealed that BrdU^+^/nestin^+^/DAPI^+^ cells accounted for 84.30% ± 6.54% among BrdU^+^ cells, which suggested that the main proliferating cells in the DG were BrdU, nestin, and DAPI three-labelled positive cells. It was to say that these proliferating cells were highly suggested NSCs, although some of these cells could be composed of other cells, such as astrocytes or others.

In the current study, we observed that the protein and mRNA expression of TLR2 in the DG increased immediately after trauma, peaked in the 3rd day, and fell gradually from the 3rd day to the 7th day but still higher than the sham level. Moreover, BrdU^+^ cells, BrdU^+^/TLR2^+^, and BrdU^+^/TLR2^+^/DAPI^+^ cells all increased in a similar way over the various time points posttrauma. More importantly, TLR2^+^/nestin^+^/DAPI^+^ three-labelled IF demonstrated that TLR2 was expressed on the NSCs in vivo.

It was accepted that NSC proliferation (often considered as BrdU^+^ cells increasing) was actually a kind of secondary events after TBI. Our results revealed that NSC proliferation was accompanied by the upregulation of TLR2 in the DG. The synchronous change of the expression of TLR2 and BrdU-positive cells posttrauma revealed that there could be a possible correlation between each other [9, 16, 30], which implied that TLR2 might play a potential role in NSC proliferation after TBI.

Recent studies indicate that endogenous neurogenesis occurs continuously throughout the whole life of all cells [31–33]. Injuries, such as trauma, stroke, inhalation injury, and also oxygen-glucose deprivation (OGD), can easily trigger neurogenesis in the brain [9, 34]. Also, trauma triggers, activates, and enhances the ability of NSCs to regenerate neurons in the DG [35]. These newborn neurons can integrate into the existed neural network and contribute to the repair process in the DG [33]. The current study based on the TBI model of rats demonstrated the pyknosis in lots of shrunken neurons in the area of the injured cortex (H&E stain). The lesions and the possibly enhanced neurogenesis in the DG (BrdU-labelled proliferating cells) coexisted. These proliferating cells maintained a higher level than sham within posttrauma in 7 days with a peak in the 3rd day. Our findings were in accordance with previous studies that reported endogenous neurogenesis in the adult brain after TBI [33].

More and more evidences showed that activated endogenous neurogenesis in the DG is essential not only for learning and memory but also for other detrimental outcomes caused by TBI [11, 36]. Nevertheless, endogenous neurogenesis of NSCs is not sufficient that is used to repair brain injury post-TBI. Therefore, strategies that effectively activate endogenous NSCs to produce more functional neural cells are needed for tissue repair and functional reconstruction posttrauma.

Recent investigations focused on the TLR2 expression on NSCs in vitro and collected the evidences on the effect of TLR2 on NSC proliferation and differentiation. Rolls et al. declared that TLR2 was expressed on NSCs in the hippocampus of the adult brain [25]. In the study of Covacu et al. [27], it was observed that the expression of TLR2 on NSCs and NSC culture, which separated from the DG in the hippocampus of the adult rat brain, was exposed on different inflammatory factors, such as IFN-*γ*, TNF-*α*, IL-1*β*, and IL-6 [37, 38]. At the same time, TLR2 mRNA was also upregulated while IFN-*γ* was put into NSC culture with supernatants from activated macrophages, but it could not be seen when TNF-*α* was put into. TLR2 agonist could activate NSCs to produce TNF-*α* in a medium, but there were no solid evidences whether there was any promotion of TLR2 agonist on the proliferation and differentiation of NSCs in vitro [39]. On the contrary, compared with NSCs separated from wild-type rats, neurogenesis around the DG from TLR2-deficiency rats was destroyed, and adult neural cell differentiation could not be seen. It is to say that TLR2 plays an important role in both proliferation and differentiation of NSCs [25, 26]. Based on existed studies, it was inferred that TLR2 activation could lead to neuronal and other neural cells' death [40] but whether the numbers of neural cells decreased needed further exploration. Moreover, because of different species of animals, different trauma models, and different functional stages of NSCs, different results should also need to be investigated.

TLR2 can also express on other cells in the brain besides NSCs, and the cells which can express TLR2 are helpful for the process of neurogenesis and inflammation in the environment of neurogenesis in the brain [11, 26, 41]. However, the growth environment of the NSC medium in vitro is different from that in vivo. Less study focused on the correlation of TLR2 expression in the DG in vivo after TBI and neurogenesis. The current study based on the TBI model helped us to observe that the level of morphology, protein, and mRNA upregulated from immediately after trauma to the 7th day in vivo after TBI [42]. It demonstrated the morphological evidences of TLR2 on NSC expression in the DG and suggested a potential correlation of TLR2 expression and NSC proliferation in the DG.

To sum up, the current experiments investigated the potential correlation between TLR2 expression and NSC proliferation in the DG in vivo. Because there was a limitation in experimental design in vivo, the results were not able to show the expression of TLR2 on the other cells, such as oligodendrocytes and neurons, in the level of protein and mRNA in the DG after trauma. Therefore, more studies are needed to precisely describe the effect of TLR2 in purer NSCs in endogenous neurogenesis in the DG after TBI. Moreover, other explorations are required to investigate the inflorescences of TLR2 to the differentiation of NSCs following the proliferation in the DG post-TBI.

## 4. Materials and Methods

### 4.1. Animals and Group

A total of 108 healthy adult male SD rats, weighing 226 ± 25 g, were purchased from the center of experimental animals of the Air Force Military Medical University. They were raised in the environment with stable temperature and humidity, 12-hour day and night cycle, and were allowed to eat and drink freely. Methods were used in order to minimize the pain and uncomfortableness of animals. Experimental procedures were in accordance with the guidelines of the National Experimental Animals, approved by the Ministry of Science and Technology of China (023915137, 09 January 2001).

An independent group design was adopted. Rats were randomly divided into the sham group (*n* = 18) and the TBI group (*n* = 90). TBI rats were divided into 5 subgroups (*n* = 18 each), sacrificed, respectively, in 6 hours postinjury, 1 day, 3 days, 7 days, and 14 days after trauma. The sham group was divided into 3 subgroups (*n* = 6 each) as controls. IF, western blotting, and real-time PCR were performed with each individual brain sample in both the TBI and sham groups.

### 4.2. TBI Model Establishment

A CCI device (Hatteras Instruments, Cary, NC, USA) was used to establish the TBI model [43]. Rats were anesthetized intraperitoneally with sodium pentobarbital (60 mg/kg) and then placed on a stereotaxic frame (Kopf Instruments, Tujunga, CA, USA). Rats were secured with ear pins and incisor bar on a frame floor. A midline scalp incision was made, and a craniotomy was performed, using a bone harvest drill with a 3.5 mm diameter, at 2 mm left to the sagittal suture and 2 mm in front of the lambdoidal suture. The bone flap was smoothly removed, and the window was in a diameter of 10 cm. A one-time injury was performed with the flat end of a metal stick with a diameter of 3 mm to contact the surface of the exposed dura. The impact parameters were as follows: 1.0 mm dura shift vertically, 100 ms contact time, and 3 m/s velocity. The scalp incision was sewed up after the operation. The above surgical procedures were done in sham rats without any injury. Animals were put on a constant temperature plate to maintain their core temperature at not below 37°C after the operation.

### 4.3. Administration of 5-Bromo-2-Deoxyuridine (BrdU)

BrdU (Sigma-Aldrich, B5002, St. Louis, Missouri, USA) was dissolved in sterile saline to the concentration of 10 mg/ml. The TBI and sham groups were injected intraperitoneally with BrdU (50 mg/kg) once a day to label NSCs in the DG in order to assess proliferation.

### 4.4. Tissue Preparation

At the established time points, rats in the TBI and sham groups were weighed and anesthetized by intraperitoneal injection of pentobarbital sodium (60 mg/kg) and then treated with heart-perfusion through the left ventricle with 100 ml 10% heparin saline for 3-5 min and 500 ml 10% paraformaldehyde phosphate buffer saline (PBS) for 2-4 h. The whole brain was removed and placed in 10% paraformaldehyde PBS (pH = 7.4) at 4°C overnight, then dehydrated in alcohol gradient and embedded in paraffin. A microtome (Leica, Nussloch, Germany) was used to cut the brain DG area into 3 *μ*m thick coronal sections containing the site of the DG which were dried and maintained at 70°C overnight. Ten coronal sections of each animal were three-labelled with BrdU/TLR2/DAPI. In order to identify NSCs in proliferating cells and verify the expression of TLR2 on NSCs after TBI, half of 10 more sections at the same plane in 6 rats in the 3-day group were prepared for to get BrdU/nestin/DAPI three-labelled treatment, and another half for nestin/TLR2/DAPI three-labelled treatment.

The TBI severity was assessed by animal behavior, such as its posture and mobility, and gross morphological change of brain tissue.

### 4.5. IF

Sections were deparaffinized by alcohol and dimethylbenzene and then incubated in citric acid and sodium citrate antigen retrieval solution (pH = 6.0) at 100°C for 100 s. The sections were incubated in hydrogen peroxide for 20 min and donkey serum for 45 min to block nonspecific signals at room temperature. The primary antibodies were used as the following to illustrate, respectively, the expression of BrdU, nestin, and TLR2: sheep anti-BrdU antibody (1 : 160, abcam, ab1893, Cambridge Science Park, Cambridge, UK), mouse anti-rat nestin antibody (1 : 100, Millipore, MAB353, Burlington, Massachusetts, USA), and mouse anti-TLR2 antibody (1 : 100, GeneTex, GTX31279, Alton Pkwy, Irvine, CA, USA). Each body was dissolved in PBS at 4°C overnight. After being washed 5 times by PBS with tween-20 (PBST), the secondary antibodies were used as follows: Alexa Fluor 594 donkey anti-sheep IgG antibody (1 : 2000, ThermoFisher SCIENTIFIC, A11016, Waltham, Massachusetts, USA), Alexa Fluor 594 donkey anti-mouse IgG antibody (1 : 2000, ThermoFisher SCIENTIFIC, A-21203, Waltham, Massachusetts, USA), Alexa Fluor 488 donkey anti-rabbit IgG antibody (1 : 2000, ThermoFisher SCIENTIFIC, A21206, Waltham, Massachusetts, USA), and Alexa Fluor 488 donkey anti-mouse IgG antibody (1 : 2000, ThermoFisher SCIENTIFIC, A21202, Waltham, Massachusetts, USA). Each body was dissolved in PBS for 3 h at room temperature. After being washed 5 times by PBST, sections were mounted with antifade mounting medium (Beyotime, P0131, Songjiang, Shanghai, China) and coverslipped. All experimental procedures are required to minimize light exposure to the tissue.

### 4.6. Microscopy and Quantification

Sections were observed by an upright fluorescence microscope (OLYMPUS, BX53, Shinjuku, Tokyo, Japan) and a high-power mercury lamp (OLYMPUS, U-RFL-T, Shinjuku, Tokyo, Japan) through the cellSens Standard system (OLYMPUS, Shinjuku, Tokyo, Japan) and photographed by a camera (OLYMPUS, DP72, Shinjuku, Tokyo, Japan).

Proliferating cells in the DG were assessed by BrdU^+^ cells in the ipsilateral DG of the injured cortex [44]. BrdU/TLR2/DAPI three-labelled fluor was used to observe BrdU^+^, TLR2^+^, and BrdU^+^/TLR2^+^ cells. Positive cells were measured in 5 consecutive fields in sections containing the DG using a fluorescence microscope. The average cell number of all 5 fields was considered as the positive cell number of each section, and the average of the positive cell number of all 5 sections was considered as the positive cell number of each brain sample of rats. Data was expressed as mean ± SEM.

The BrdU^+^/nestin^+^/DAPI^+^ three-labelling was used to show proliferating NSCs in the DG after TBI. The percentage of BrdU^+^/nestin^+^/DAPI^+^ cells in BrdU^+^ cells was counted, and nestin^+^/TLR2^+^/DAPI^+^ three-labelling were taken to analyze the expression of TLR2 in possible NSCs.

### 4.7. Western Blotting

At each time point, the rats' brains were removed immediately after the rats were sacrificed by excessively intraperitoneal anesthesia. Hippocampal tissues were separated as quickly as possible on ice and then dissolved in a buffer in homogenizers, which contained RIPA lysis buffer (Beyotime, P0013, Songjiang, Shanghai, China) and phenylmethylsulfonyl fluoride (Beyotime, ST506, Songjiang, Shanghai, China). The above solution was put around the ice for 30 min and then centrifuged for 20 min in 15000 rpm at 4°C. Supernate was taken for protein quantification through the Bicinchoninic Acid Protein Assay kit (Beyotime, P0010, Songjiang, Shanghai, China). Protein samples were added with loading buffer and boiled at 100°C for 10 min. Samples containing 40 *μ*g protein were used for gel electrophoresis, and then, the protein on the gel was transferred to nitrocellulose membranes. Primary antibodies were used as follows: mouse anti-TLR2 antibody (1 : 500, Biorbyt, orb191498, San Francisco, CA, USA) and mouse anti-*β*-actin antibody (1 : 3000, TDY BIOTECH, TDY041, Beijing, China) overnight at 4°C. After washed 4 times by PBST, membranes were incubated with HRP- (horseradish peroxidase-) Conjugated Goat anti-Mouse IgG (H+L) antibody (1 : 40000, TDY BIOTECH, S001, Beijing, China) for 1 h in room temperature. The protein bands were observed through Western LumaxLight Superior (ZETA LIFE, 310208, USA), and the greyscale of TLR2 to *β*-actin was considered as the expression of TLR2.

### 4.8. Real-Time PCR

At each time point, rats were sacrificed in the way as used above. The tissue of the DG was removed. Total RNA was extracted by the MiniBEST Universal RNA Extraction Kit (TaKaRa, 9767, Toyko, Japan). Prime Script™ one-step RT-PCR Kit Ver.2 (TaKaRa, RR055, Toyko, Japan) was used to synthesis cDNA. TB Green™ Fast qPCR Mix (TaKaRa, RR430, Toyko, Japan) was used in the CFX96 real-time PCR detection system (Bio-Rad, Hercules, CA, USA). GAPDH was adopted as the endogenous reference gene for the expression of the TLR2 gene.

The sequences of primers were as follows (Table 1): TLR2 forward: 5′-TGGAAGCAGGTGACAACC-3′; TLR2 reserve: 5-ACCTTCGTCCACTGTTGG-3; GAPDH forward: 5-ACAGCAACAGGGTGGTGGAC-5; GAPDH reserve: 5-TTTGAGGGTGCAGCGAACTT.

### 4.9. Statistical Analysis

All of the data was expressed as mean ± SEM. Multiple comparisons in TLR2 and proliferation-related factors in the DG were analyzed using one-way ANOVA followed by post hoc Bonferroni's test using SPSS 22.0.0 and GraphPad prism (v.7.0.1, GraphPad software, San Diego, CA, USA). The *p* < 0.05 was considered to be statistically significant.

## 5. Conclusion

The current study illustrated that BrdU^+^ and nestin^+^ cells were TLR2^+^ cells in the DG after trauma through the TBI model of rats, indicating that obvious cell proliferation (considered as newly NSC formation) was followed with an increased expression of TLR2. It suggested that NSC proliferation might be correlated with TLR2 upregulation in the DG and TLR2 might play a potential role in endogenous neurogenesis in the DG after TBI. The results demonstrated that there was evident NSC proliferation in the DG. Because newborn neurons and other cells grew mixed in the process of NSC differentiation, specific markers should be used to identify cell types and their possible origin, which was the goal of future investigations.

## Figures and Tables

**Table 1:** Gene sequences for primer synthesis.

Gene	Primers	Sequence 5′-3′
TLR2	Sense	TGGAAGCAGGTGACAACC
	Antisense	ACCTTCGTCCACTGTTGG
GAPDH	Sense	ACAGCAACAGGGTGGTGGAC
	Antisense	TTTGAGGGTGCAGCGAACTT

## Data Availability

All data generated or analyzed during this study are included in this article.
